# In vivo antiplasmodial activity and toxicological assessment of hydroethanolic crude extract of *Ajuga remota*

**DOI:** 10.1186/s12936-017-1677-3

**Published:** 2017-01-13

**Authors:** Aschalew Nardos, Eyasu Makonnen

**Affiliations:** 1 Pharmacology Unit, School of Medicine, Hawassa University, P O Box 1560, Hawassa, Ethiopia; 2 Department of Pharmacology, School of Medicine, Addis Ababa University, Addis Ababa, Ethiopia

**Keywords:** Malaria, *Ajuga remota*, *Plasmodium berghei* (ANKA strain)

## Abstract

**Background:**

Malaria is one of the most life-threatening health problems worldwide and treatment has been compromised by drug resistance. Identifying lead molecules from natural products might help to find better anti-malarial drugs, since those obtained from natural sources are still effective against malarial parasites. This study aimed at investigating the in vivo antiplasmodial activity of crude extract of the leaves of *Ajuga remota* together with its safety in mice models.

**Methods:**

In vivo parasite growth inhibitory effect of crude extract was assessed in mice inoculated with *Plasmodium berghei* (ANKA strain). The in vivo antiplasmodial activity of the test extract was performed against early infection (4-day suppressive test), curative effect against established infection and prophylactic effect against residual infection. Acute and sub-acute toxicity were carried out according to OECD guidelines.

**Results:**

In vivo parasite growth inhibition effect of hydroethanolic crude extract of *A. remota* was evaluated at 30, 50 and 100 mg/kg dose levels. It suppressed parasitaemia by 77.34% at 100 mg/kg dose level in the 4-day test. In curative and prophylactic potential tests, it suppressed parasitaemia by 66.67 and 59.66% at 100 mg/kg dose level, respectively. In vivo toxicity tests revealed no toxicity. All parasitaemia suppressions were statistically significant at P < 0.05 as compared to the vehicle-treated group. The crude extract also prolonged survival time in a dose dependent manner.

**Conclusions:**

The investigation results suggest that the leave extract of *Ajuga remota* possesses antimalarial activity.

## Background

Malaria is one of the major infectious diseases responsible for the high rate of mortality and morbidity in developing countries. It is caused by Apicomplexan protozoan parasites of the genus *Plasmodium* [1]. The disease burden is very alarming in the sub-Saharan countries and mainly affects children less than five years of age, which accounts for about 78% of all global deaths [2]. Malaria also causes series adverse effects during pregnancy for both mother and infant, such as maternal anaemia, low birth weight, and increased perinatal and infant mortality [3]. This infectious disease is highly fatal unless diagnosed and treated in a timely fashion. It may also predisposes the patient to opportunistic infections [4].

*Ajuga remota* belongs to a genus of about 40–50 species of annual and perennial herbaceous flowering plants in the Mint (*Lamiaceae*) family [5, 6]. The aqueous or alcohol extract of fresh or dried leaves of *A. remota* have been traditionally used for the treatment of diabetes, malaria, pain and fevers, toothache, skin disease, hypertension, stomach ache, pneumonia, liver problem and swelling of legs [7, 8]. Various compounds have been isolated from the leaves of *Ajuga remota,* including five different neo-clerodane diterpenes [8, 9]. Sterols (ajugalactone), triterpenoid (ergosterol-5, 8-endoperoxide and iridoid glycoside (8-O-acetylharpagide) were also isolated from the plant, as well as phenolic acids, flavonoids and saponins [8, 10]. The plant has wide traditional medicinal applications, and the present study was designed to confirm its anti-malarial efficacy and safety of its use.

## Methods

### Plant material preparation

Fresh leaves of *A. remota* (Labiatae) were collected in October 2012 around Entoto area, North of Addis Ababa. The leaves of the plant material were identified by a taxonomist at the National Herbarium, College of Computational and Natural Sciences, Addis Ababa University. A voucher number of AR-001 was given and deposited at the herbarium for future reference. The study was performed after obtaining ethical approval from institutional review board of the College of Health Sciences, Addis Ababa University.

### Extraction

The leaves were air dried at room temperature under shade and ground to a powder using pestle and mortar. A total of 250 g dried leaves were macerated in 80% of 2.5-l ethanol (1:10 w/v) for 72 h. The extraction was carried out by using an orbital shaker at 120 rpm. The mixture was first filtered with gauze and then Whatman filters paper No. 1 (Whatman^®^, England). The mark was re-macerated for another 72 h twice and filtered. The combined ethanol filtrates were then concentrated by rotary evaporator (Buchi Rota vapor, Switzerland) at a temperature of 40 °C. The residual water was removed by lyophilizer (operon, Korea vacuum limited, Korea) at −44 °C. The extract was dark green lustrous gummy substance. After drying, a total of 27.32 g of dry extract was collected. The extract was stored in tightly closed bottle container in a refrigerator at −20 °C until used [11].

### In vivo toxicity assay

#### Acute toxicity test

The hydroethanolic crude extracts of *A. remota* was tested in non-infected female Swiss albino mice aged 6–8 weeks and weighing 23–31 g. For each crude extract, 10 mice were used by randomly dividing them into two groups of 5 mice per group. The mice were starved for 3 h before the experiment began and only allowed water ad libitum. After 3 h, the extract was administered orally in a single dose. The mice in group I was given 0.2 ml of 2000 mg/kg body weight of the extract. The mice in the control groups received 0.2 ml of the vehicle (distilled water) used for dissolving the extract. Then, the mice were observed continuously for 1 h, followed by 4 h observations for 24 h and thereafter daily for 14 days, for any manifestation of toxicity [12].

#### Sub-acute toxicity test

For sub-acute toxicity studies, weight and haematological parameter like packed cell volume (PCV) were determined before and after treatment. PCV was determined by micro-haematocrit reader (Hawksley and Sons, England). The extracts in each case were administered orally for 4 days (i.e., D0 to D3). The mice were grouped randomly, five mice per group. The mice in group I, II and III were given 500, 750 and 1000 mg/kg body weight in single dose volume of 0.2 ml of each crude extract, respectively. The control group received 0.2 ml of dH_2_O. The data were recorded to check for reduction in PCV and weight losses on day 0 and 3 (12 h after administration of the last dose) and thereafter, the mice were closely observed for 1 month [12].

### In vivo antiplasmodial activity assay

#### Parasite inoculation

Albino mice earlier infected with *Plasmodium berghei,* ANKA strain, (parasitaemia level of 20–30%) were used as donor. The donor mice were then sacrificed with ethyl ether anesthesia and blood was collected by cardiac puncture into heparinized vacutainer tube. The blood was then diluted with physiological saline (0.9%) based on parasitaemia level of the donor mice and the red blood cell (RBC) count of normal mice, in such a way that 1 ml blood contains 5 × 10^7^ infected RBC [13]. Each mouse was then given 0.2 ml of this diluted blood intraperitoneally, which contained 1 × 10^7^ *P. berghei* infected RBCs.

### Grouping and dosing of animals

#### Four-day suppressive test

This test was used to evaluate the schizontocidal activity of the crude extract against *P. berghei* infected mice [14]. Antiplasmodial activity of the test extract was performed in a 4-days suppressive standard test. Male Swiss Albino mice weighing 22–29 g were inoculated on the first day (Day 0), intraperitoneally, with 0.2 ml of infected blood. The mice were then divided randomly into five groups of six mice per group. Three groups (II, III and IV) were assigned as test groups whereas the other two groups (I & V) were used as control (negative and positive) groups. Three hours after infection 30, 50 and 100 mg/kg/day of hydroalcoholic crude extract of *A. remota* were administered to the test groups. Chloroquine at the dose of 25 mg/kg/day and an equivalent volume of vehicle (0.2 ml 7% tween 80 solution) were administered to the positive and negative control groups, respectively, for four consecutive days (Day 0–3). On the fifth day (on day 4, 24 h after the last dose i.e. 96 h post-infection), thin blood smears were made from the tail of each mouse, fixed with methanol and stained with 10% Giemsa. The parasitaemia level was determined by counting the number of parasitized erythrocytes out of 100 erythrocytes in random 8 fields of microscope.

Parasitaemia was determined by light microscopy using a 100× objective lens and the following equation:

$$ \% {\text{ Parasitemia}}\, = \,\frac{\text{Number of Parasitized RBC}}{\text{Total Number of RBC Counted}}\, \times \, 100 $$

Average percentage chemo-suppression was calculated as

$$ 100\left[ {\frac{{{\text{A}} - {\text{B}}}}{\text{A}}} \right] $$

where A is the average percentage parasitaemia in the negative control group and B is the average percentage parasitaemia in the test group. The capability of the various doses of the crude extract in preventing body weights and packed cell volume (PCV) reduction of the mice as a result of raise in parasitaemia were followed. The weight, PCV and rectal temperature were taken at D_0_ and D_4_.

### Test for curative activity (Rane’s test)

The chemotherapeutic activity of hydroethanolic crude extract was carried out in established infection [15]. On the first day (Day 0), 0.2 ml standard inocula of 1 × 10^7^ *P. berghei* infected erythrocytes were inoculated in mice intraperitoneally. Seventy-two hours later (Day 3), mice were randomly divided in three test groups (II, III and IV) of six mice each. Group I and V were assigned as negative and positive controls, respectively. Then, the test groups were dosed with 30, 50, 100 mg/kg/day of the extract. Negative and positive controls received vehicle (0.2 ml 7% tween 80) and chloroquine 25 mg/kg/day orally, respectively. Giemsa-stained thin blood film was prepared from the tail of each mouse on days 3 and 7 to monitor parasitaemia level.

### Test for prophylactic activity

The prophylactic activity of the extract was tested using the residual infection procedure [16]. On the first day (Day 0), mice were randomized into five groups six mice per group. Groups II, III, IV were used for evaluation of test samples. Group I and V were used as negative and positive controls, respectively. Group II, III and IV received oral doses of 30, 50 and 100 mg/kg/day for three consecutive days (Day 0–2). Whereas negative and positive controls received vehicle (0.2 ml 7% tween 80) and chloroquine 25 mg/kg/day orally, respectively. On the fourth day, all mice were infected with 0.2 ml standard inocula of 1 × 10^7^ *P. berghei* infected erythrocytes intraperitoneally and 72 h later thin blood films were prepared to determine the level of parasitaemia.

### Determination of packed cell volume

Packed cell volume (PCV) was measured to predict the effectiveness of the test extract in preventing haemolysis resulting from increased parasitaemia. Heparinized capillary tubes were used for collection of blood from tail of each mouse. The capillary tubes were filled with blood up to 3/4th of their volume and sealed at the dry end with sealing clay. The tubes were then placed in a micro-haematocrit centrifuge (Gelma Awhksley, England) with the sealed end outwards and centrifuged for 5 min at 12,000 rpm. The tubes were then taken out of the centrifuge and PCV was determined using a standard micro-haematocrit reader. PCV is a measure of the proportion of RBCs to plasma and measured before inoculating the parasite and after treatment using the following relationship:

$$ {\text{PCV}}\, = \,\frac{\text{Volume of Erythrocytes in a Given Volume of Blood}}{\text{Total Blood Volume}} $$

### Monitoring body weight

In the 4-day suppressive test, body weight of each infected mice was measured before infection on day 0 and after treatment on day 4. However, for Rane’s test the body weight was measured after infection on day 3 and after treatment on day 7. The body weight of each mouse was measured using a sensitive digital weighing balance (Mettler Toledo, Switzerland).

### Determination of rectal temperature

In a 4-day suppressive test, the body temperature of each mouse was determined before infection on day 0 and after treatment on day 4 using digital thermometer.

### Monitoring mean survival time

In all of the in vivo antiplasmodial models, mortality was monitored and the number of days from the time of inoculation of parasite up to death was recorded for each mouse in the treatment and control groups for 30 days. The mean survival time (MST) for each group was calculated as follows:

$$ {\text{MST}} = \frac{{\text{Sum of Survival Time of All Mice in a Group}}\, ( {\text{days}} ) } {{\text{Total Number of Mice in the Group}}} $$

### Data analysis

Data were analysed using Graph Pad Prism v 6.02. Comparisons were made among negative and positive controls as well as treatment groups using a one-way analysis of variance (ANOVA) followed by Tukey’s multiple comparison tests. Mean PCV, rectal temperature and body weight before and after infection and treatment were compared by two tailed paired *t* test. The result was considered statistically significant at 95% confidence level and P < 0.05.

## Results

### Acute oral toxicity assessment

In the in vivo acute toxicity studies of the plant extract, there were no gross physical and behavioural changes such as rigidity, sleep, diarrhoea, depression, abnormal secretion and hair erection for 24 h. All the mice survived within the 2-week observation period.

### Sub-acute oral toxicity assessment

The direct toxic effects of the crude extract on body weight and blood parameter (PCV) in healthy mice were evaluated by administering higher doses of the extract for 4 days. All hydroethanolic crude extracts of the three plants showed no statistically significant reduction in PCV on day 3 as compared to day 0 (Table 1) in non-infected normal mice. In any of the doses, no mortality and insignificant difference in body weight was seen. However, significant (P < 0.05) body weight gain was observed with 500 mg/kg of *A. remota.*

**Table 1:** Sub-acute toxic effects of hydroethanolic crude extracts of *Ajuga remota*

Treatment	Dose (mg)	Parameters	Day 0	Day 3
Crude extract	NC	Body weight (g)	28.8 ± 0.37	30.20 ± 1.28
		PCV (%)	51.00 ± 2.92	51.20 ± 1.59
	500	Body weight (g)	27.32 ± 0.21	28.40 ± 0.24*
		PCV (%)	49.40 ± 0.24	48.92 ± 0.69
	750	Body weight (g)	28.18 ± 0.67	27.70 ± 0.95
		PCV (%)	51.90 ± 1.49	51.16 ± 1.44
	1000	Body weight (g)	31.04 ± 0.99	29.28 ± 1.57
		PCV (%)	49.94 ± 0.45	49.82 ± 0.83

Results are expressed as mean ± SEM (n = 5)

*NC* negative control, *PCV* packed cell volume

* There is significant difference at P < 0.05 between day 0 and day

### In vivo assays

#### Four-day suppressive effect

**Fig. 1:**
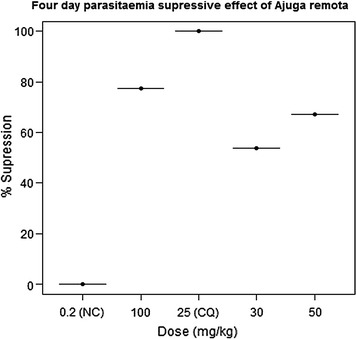
Four day parasitaemia suppressive effect of different doses of crude extract of *Ajuga remota* as compared to the negative control (NC) and the standard treatment, chloroquine (CQ)

The percentage suppression analysis of the extract showed decrease (P < 0.05) in parasitaemia at all dose levels as compared to the negative control group. The group received 100 mg/kg of the extract exhibited maximal suppression (77.54%) of parasitaemia than the other groups (Fig. 1). All doses of the extract significantly enhanced the survival time of the mice in a dose dependent manner as compared to the negative control group; though the effect was significantly lower than the group which received chloroquine (Table 2).

**Table 2:** Average parasitaemia and mean survival time of hydroethanolic crude extracts of *Ajuga remota*

Treatment	Dose (mg/kg)	% Parasitaemia	Survival time (days)
Crude extract	NC	32.15 ± 0.66	7 ± 0.86
	30	14.82 ± 0.98*^,a,c,d^	11 ± 1.13*^,a^
	50	10.59 ± 0.69*^a,b,d^	17 ± 1.00*^a^
	100	7.22 ± 0.57*^,a,b,c^	18 ± 0.73*^,a^
	CQ	0.00 ± 0.00*^,a,b,c,d^	29 ± 0.73*^,a^

% parasitaemia and survival time results presented as mean ± SEM; n = 6

NC, negative control; CQ, chloroquine

*  Values are significantly different at P < 0.05

^a^Compared to negative control

^b^Compared to 30 mg/kg

^c^Compared to 50 mg/kg

^d^Compared to 100 mg/kg

### Effect on body weight, PCV and temperature

Hydroethanolic crude extract of *A. remota* prevented significant reduction of body weight on day 4 as compared to day 0 at all dose levels. On day 4, the percent change in body weight increment seemed almost dose dependent. In addition to that, the crude extract averted significant loss of PCV between days 0 and 4 at all dose levels. The negative control group showed significant loses of body weight and PCV (P < 0.05) on day 4. In the chloroquine treatment group, no significant change in both weight and PCV was observed (Table 3).

**Table 3:** Effect of hydroethanolic crude extract of *Ajuga remota* on body weight and PCV of *Plasmodium berghei* infected mice in 4 day suppressive test

Treatment	Dose (mg/kg)	Body weight in grams			Packed cell volume		
		Day 0	Day 4	% ∆W	Day 0	Day 4	% ∆PCV
Crude extract	NC	23.0 ± 1.09	21.0 ± 1.55*	−8.69	54.0 ± 2.61	49.0 ± 1.58*	−9.26
	30	24.1 ± 1.61	23.3 ± 1.88	−3.32	53.0 ± 1.47	51.0 ± 4.16	−3.77
	50	22.0 ± 0.89	22.3 ± 1.17	1.36	54.0 ± 6.07	52.33 ± 7.05	−3.09
	100	23.2 ± 1.39	23.7 ± 0.93	2.16	52.0 ± 6.17	51.33 ± 2.94	−1.28
	CQ	25.0 ± 1.77	25.8 ± 0.91	3.20	51.0 ± 2.41	50.83 ± 2.35	−0.33

Results presented as mean ± SD; n = 6

% ∆W, increment in weight; % ∆PCV, % increment in packed cell volume

* Significant difference between Day 0 and Day 4 (P < 0.05)

**Fig. 2:**
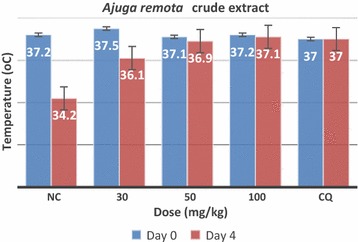
The effect of hydroethanolic leaf extract of *Ajuga remota* on rectal temperature of *P. berghei* infected mice between day 0 and 4 in suppressive test. *NC* negative control, *CQ* chloroquine

Analysis of rectal temperature of *P. berghei* infected and treated mice indicated that crude extract of *A. remota* prevented significant loss of body temperature at 50 and 100 mg/kg dose levels on day 4. However, 30 mg/kg dose level of the crude extract; could not avert loss of body temperature on day 4. The control group treated with vehicle showed significant (P < 0.05) loss of temperature on day 4. But, the group treated with the standard drug, chloroquine maintained their temperature on day 4 (Fig. 2).

### Curative effect (Rane’s Test)

**Fig. 3:**
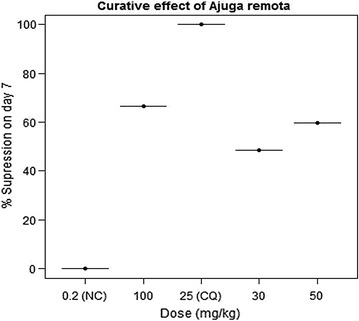
Curative effect of different doses of crude extract of *Ajuga remota* on day 7 as compared to the negative control (NC) and the standard treatment, chloroquine (CQ)

In curative potential evaluation of *A. remota* crude extract, all the three doses significantly reduced (P < 0.05) parasitaemia level on day 7 as compared to day 3. Parasitaemia reduction showed a dose dependent trend relative to the vehicle. On day 7, the highest parasitaemia suppression of 66.67% was seen by 100 mg/kg dose of the extract (Fig. 3). Survival time prolongation was observed at 50 and 100 mg/kg of the extract. No apparent survival time difference was observed between the group treated with vehicle and 30 mg/kg of the extract. Chloroquine-treated group showed total eradication of the established *P. berghei* infection on day 7. However, no curative effect was shown by any of the test group treated with different doses of the extract unlike chloroquine (Table 4).

**Table 4:** Average parasitaemia and mean survival time of hydroethanolic crude extract of *Ajuga remota* in the Rane’s Test

Treatment	Dose (mg/kg)	Average % parasitaemia		Survival time
		Day 3	Day 7	
Crude extract	NC	19.33 ± 1.88	33.60 ± 0.97	7.33 ± 0.56
	30	21.67 ± 1.53	17.33 ± 0.80*	7.83 ± 1.01
	50	24.40 ± 1.93	13.50 ± 0.69*	13.00 ± 0.37*
	100	23.50 ± 2.84	11.20 ± 0.35*	16.67 ± 0.76*
	CQ	22.70 ± 0.73	0.00 ± 0.00*	29.00 ± 0.58*

% parasitaemia and survival time results presented as mean ± SEM; n = 6

NC, negative control; CQ, chloroquine

* Values are significantly different at P < 0.05 between day 3 and day 7

**Fig. 4:**
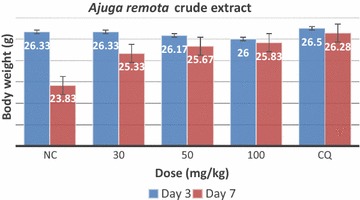
The effect of hydroethanolic crude extract of *Ajuga remota* on body weight of *P. berghei* infected mice in curative test. *NC* negative control, *CQ* chloroquine

In evaluating effect of *A. remota* on body weight in the curative test, there was significant body weight loss (P < 0.05) at 30 and 50 mg/kg doses of the extract on day 7 as compared to day 3. The 100 mg/kg dose of the extract prevented significant loss of body weight on day 7. Vehicle administered group showed significant loss of body weight. On the contrary, chloroquine treated group did not exhibit significant weight loss (Fig. 4).

### Prophylactic effect

**Fig. 5:**
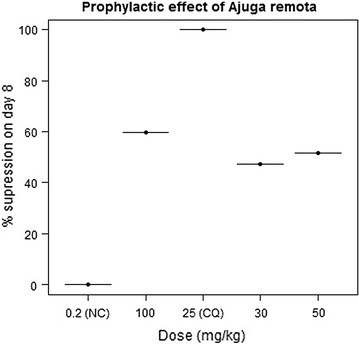
Parasitaemia suppressive effect of different doses of crude extract of *Ajuga remota* on day 8 as compared to the negative control (NC) and the standard treatment, chloroquine (CQ) in the prophylactic test

In the residual test, the activity of the extract was significant on day 8 (P < 0.05), at the tested doses of 30, 50 and 100 mg/kg/day. They produced 47.22, 51.53 and 59.66% suppression of parasitaemia, respectively (Fig. 5). However, significant prolongation of survival time was observed at the highest dose of the extract (100 mg/kg/day) (Table 5).

**Table 5:** Average parasitaemia and survival time of hydroethanolic crude extract of *Ajuga remota* in *P. berghei* infected mice in prophylactic test

Treatment	Dose (mg/kg)	Average % parasitaemia	Survival time (Days)
		Day-8	
Crude extract	NC	36.32 ± 1.57	7.83 ± 0.17
	30	19.17 ± 1.28*^,a^	8.83 ± 0.48
	50	17.60 ± 1.53*^,a^	9.18 ± 0.31
	100	14.65 ± 0.64*^,a^	10.33 ± 0.67*
	CQ	0.00 ± 0.00*^,a,b,c,d^	28.67 ± 0.56*

% parasitaemia and survival time results presented as mean ± SEM; n = 6

NC, negative control; CQ, chloroquine

*  Values are significantly different at P < 0.05

^a^Compared to negative control

^b^Compared to 30 mg/kg

^c^Compared to 50 mg/kg

^d^Compared to 100 mg/kg

## Discussion

*Ajuga remota* is widely applied in the folkloric medicine of Ethiopia for treating various types of disorders [17]. Plant medicines has been utilized by majority of the world population for primary health care [18]. However, lack of validated information has been a major concern with respect to the use of plant medicines [19].

The results of the acute toxicity of *A. remota* revealed that there was no mortality observed up to the maximum dose level of 2000 mg/kg body weight of the extract administered orally, where single high dose is recommended for testing acute toxicity [12]. No behavioural changes were observed either viz apathy and reduced locomotors activity. Accordingly, 2000 mg/kg of each plant extract was found safe, *i.e*., the approximate median lethal dose (LD_50_) of the extracts in the experimental mice was higher than 2000 mg/kg. The results observed in the acute toxicity study with *A. remota* is in agreement with those of the previous study [20]. The reduction in body weight gain is a simple and sensitive index of toxicity after exposure to toxic substances [21]. In sub-acute toxicity study, all the groups treated with 500, 750 and 1000 mg/kg doses of the crude extract did not show significant change in weight on day 4 as compared to day 0 except the group received 500 mg/kg of the extract which showed significant (P < 0.05) body weight gain on day 4. This observation indicates that the crude extracts may not contain appetite stimulant compounds [22]. Study on haematological parameters is important to discriminate the toxic effects of exogenous compounds on animals [23]. In this study, PCV, an index of anaemia, did not show significant difference on day 4 as compared to day 0 of either treated or untreated mice. This might indicate the safety nature of the extract.

In vivo models are usually applied in antimalarial studies for they allow the possible prodrug effect and likely boosting of the immune system in eradication of the infectious agent [24]. In the four-day parasitaemia suppression study, treatment of mice with crude extract of *A. remota* reduced the erythrocytic stage development of *P. berghei*. Parasitaemia was suppressed in a dose dependent manner indicating that the plant has antimalarial activity. Similar results were obtained in studies reported from the same species of *A. remota* [25]. The parasite suppressive effect of plant extract might be through indirect boosting of the immune system or by inhibition of other target pathways which are not fully realized [26]. Likewise, the parasite inhibitory effect of the extract with unknown compounds might be attributed to the antiplasmodial activity of specific compound or group of compounds [27]. *Ajuga remota* possesses compounds such as, terpenoids, sterols, flavonoids and saponins that might be responsible for its antiplasmodial activity [8–10].

The mean survival time is important to evaluate the anti-malarial activity of plant extracts [28]. The extract prolonged survival time of mice at all dose levels which is associated with suppression of parasitaemia. Haematological abnormalities like anaemia, body weight loss and temperature reductions are common characteristics of *P. berghei* infected mice [29]. Plants with anti-malarial activity are expected to prevent body weight loss in infected mice resulting from rise in parasitaemia. The crude extract of *A. remota* significantly prevented body weight loss in a dose dependent manner on day 4 as compared to day 0. *Plasmodium berghei* infected mice showed decrease in metabolic rate before death which as a result caused drop in internal body temperature [30]. Thus, plant extracts with anti-malarial activity must prevent rapid falling of body temperature [29]. Crude extract of *A. remota* prevented body temperature drop at 50 and 100 mg/kg doses on day 4 as compared to day 0.

Rodent malaria causes parasite-induced fall of PCV, which occurred approximately 48 h post-infection [29]. *Plasmodium berghei* infected mice suffer from anaemia because of erythrocyte destruction, either by parasite multiplication or by spleen reticuloendotelial cell action as the presence of many abnormal erythrocytes stimulates the spleen to produce many phagocytes [22]. All of these mechanisms are accountable to malaria induced anaemia both in mice and men [31]. It was noted that crude extract of *A. remota* prevented reduction in PCV at all dose levels in between day 0 and day 4. Though *A. remota* contains saponins [8, 10] responsible for haemolytic effects, the sub-acute toxicity study done in healthy non-infected mice confirmed that the crude extract did not cause haematological abnormalities (PCV reduction). PCV reduction and body weight loss preventive effect of the hydroethanolic crude extract of *A. remota* in infected mice is consistent with the sub-acute toxicity study carried out in healthy non-infected mice. This confirms that the plant extract has no impact on the measured parameters. The preventive effect of haematological abnormalities (PCV reduction), body weights lose and rectal temperature drop by the crude extract of *A. remota* infer its antimalarial activity.

In the curative test, crude extract of *A. remota* did not eradicate parasites completely on day 7. However, it showed parasite suppressive effects. Complicated syndromes of malaria comprise of many inflammatory mediators which may enhance cell to cell interaction (cytoadherence), cell stimulation through malaria-derived antigens and host-derived factors like cytokines. Cytokines are also responsible to cause fever in the host [32]. The curative antiplasmodial properties of *A. remota* may be due to the inhibition of the production and/or release of these inflammatory mediators associated with malaria. Surprisingly, *A. remota* is traditionally used for its pain and fever reduction ability [7, 8, 33].

In the prophylactic assay, *A. remota* resulted in better efficacy than the negative control on day 8. The probable mechanism to produce prophylactic activity on *P. berghei* infection might be inhibiting proliferation of parasites due to direct cytotoxic effect [34] and modulation of the membrane of the erythrocytes preventing parasite invasion [35].

In vivo antiplasmodial activity can be classified as moderate, good, and very good if an extract displayed percentage parasitaemia suppression equal to or greater than 50% at a dose of 500, 250 and 100 mg/kg body weight per day, respectively [36]. Based on this classification, the crude extract of *A. remota* showed very good antiplasmodial activity below 100 mg/kg/day dose level. Similarly, four day suppressive in vivo evaluation of the wet and dried leaf aqueous extracts of *A. remota* showed 90.35 and 82.82% suppression of parasitaemia, respectively [25].

## Conclusions

This investigation confirmed that *A. remota* has anti-malarial activity in the in vivo model. The results of the present study, therefore, likely support the traditional use of this plant in Ethiopia.

## Abbreviations

dH_2_O: distilled water
DMSO: dimethyl sulfo oxide
IC_50_: 50% inhibitory concentration
MTT: methyl thiazol tetrazolium
PBS: phosphate buffer solution
PCV: packed cell volume
RBC: red blood cell
WHO: World Health Organization
